# Integrating Artificial Intelligence Into Health Informatics and Information Education: A Competency-Based Framework Using Miller’s Pyramid

**DOI:** 10.63116/ahisp-25-004

**Published:** 2026-04-17

**Authors:** Shannon H. Houser, Cathy A. Flite, Susan L. Foster, Angela Morey

**Affiliations:** 1 Health Services Administration, University of Alabama at Birmingham; 2 Health Services Administration and Policy, Temple University; 3 Health Information Management, Florida SouthWestern State College; 4 Health Administration Department, University of North Georgia

**Keywords:** artificial intelligence, health informatics and information education, educational frameworks, Miller’s Pyramid, competency-based learning, AI-enhanced learning, responsible AI in education

## Abstract

The integration of artificial intelligence (AI) into health informatics and information management (HIIM) education presents both transformative opportunities and ethical complexities. As health care systems increasingly adopt AI-enabled tools and workflows, educational programs must prepare students to not only understand AI technologies but also apply them responsibly and effectively. In this article, the authors propose a competency-based framework for embedding AI into HIIM curricula, using Miller’s Pyramid of Clinical Competence to scaffold progressive learning outcomes. With this developmental model, educators guide students from foundational knowledge to applied reasoning, simulation-based practice, and real-world performance using AI tools. The model can be used to foster problem-solving abilities and support workforce readiness by aligning AI-enhanced assignments with core HIIM domains. Ethical considerations are embedded throughout the learning process to promote the responsible and equitable use of AI. By offering flexible guidance for integrating AI into diverse educational settings, the competency-based framework enables instructors to design competency-aligned learning experiences that foster technical proficiency, ethical awareness, and professional confidence in today’s AI-driven health care environment.

## Introduction

The rapid integration of artificial intelligence (AI) into health care is transforming how clinical data are captured, curated, analyzed, and applied. AI now supports diagnosis, documentation, decision support, predictive modeling, and patient engagement—functions once performed exclusively by humans are now increasingly augmented or automated by intelligent systems.^1^ This evolution presents not only immense opportunities but also new responsibilities for health informatics and information professionals, who are expected to steward the data life cycle while upholding principles of transparency, equity, and governance.

As AI becomes embedded in electronic health records (EHRs), coding software, quality improvement platforms, and patient portals, the demand grows for professionals who are fluent in both the capabilities and consequences of AI. Graduates of health informatics and information management (HIIM) programs must be prepared to not just use AI tools but also evaluate them critically—understanding what they do, how they work, where they fall short, and how they may affect safety, equity, privacy, and clinical integrity.

Recent scholarship urges educators to go beyond technical or prompt engineering training. Students must learn to interrogate AI tools, critique outputs, and evaluate potential biases, blind spots, and downstream risks.^1^ Effective AI integration requires a longitudinal, iterative approach—one that is embedded across the curriculum and not relegated to a single course.^2^^,^^3^

Despite these imperatives, many HIIM curricula remain misaligned with the performance-based expectations of modern AI practice. Programs often emphasize conceptual knowledge—definitions, typologies, and examples—without scaffolding toward applied reasoning, simulation-based practice, or supervised performance in authentic settings. This creates a persistent “knowing–doing” gap: Students may understand AI terminology yet lack readiness to apply or evaluate AI tools in workflows such as coding audits, de-identification, documentation quality checks, or algorithm governance. Miller’s long-standing critique of overreliance on knowledge testing remains highly relevant—true competence is evidenced by not only what learners know but also what they do in workplace-like conditions.^4^^,^^5^

Accreditation standards are also shifting toward demonstrable competencies and real-world readiness. The 2026 Commission on Accreditation for Health Informatics and Information Management Education (CAHIIM) Health Information Management Accreditation Standards now emphasize applied learning in analytics, emerging technologies, governance, and privacy/security.^6^ Likewise, international frameworks such as the Healthcare Information and Management Systems Society Technology Informatics Guiding Education Reform (TIGER) initiative—which provides internationally validated, role-based informatics competencies—can be adapted to support AI-infused, future-ready health informatics education.^7^

To address these challenges, in this article, the authors present a practical, staged framework for integrating AI into HIIM curricula using Miller’s Pyramid of Clinical Competence. The model aligns AI learning outcomes and assessments with progressive levels of cognitive and professional development—Knows, Knows How, Shows How, and Does—to support performance-based, accreditation-aligned education.^4^ Concrete examples of assignments, tools, ethical considerations, and assessment strategies are offered to help educators build curriculum pathways from AI literacy to trustworthy, practice-ready implementation.

## Foundational Theory of Miller’s Pyramid

Introduced by George E. Miller in 1990, Miller’s Pyramid of Clinical Competence has become a foundational model in health professions education.^4^ It outlines a progression from acquiring knowledge (Knows), to applying it in context (Knows How), to demonstrating it in structured environments (Shows How), and to ultimately performing it in real-world settings (Does). This framework remains powerful in guiding the design of learning activities and assessments that evolve in complexity, authenticity, and evaluative rigor.

For AI education in HIIM, Miller’s Pyramid provides a natural pedagogical structure. AI concepts must be not only understood but also applied, demonstrated, and implemented in authentic environments. For instance, recognizing algorithmic bias (Knows) is insufficient without the ability to critique AI outputs (Knows How), simulate real-world workflows (Shows How), and ultimately guide safe AI use in documentation review or system governance (Does).

### The Four Levels Applied to AI Education

- **Knows:** Students build foundational understanding of AI models, data provenance, bias, and relevant legal frameworks. Assessments may include quizzes, concept maps, and case-based scenarios in which students identify ethical or regulatory issues.^1^
- **Knows How:** Learners apply knowledge by selecting AI tools, analyzing outputs, crafting prompts, or developing governance strategies. Case studies, prompt audits, and error analysis assignments support applied reasoning.^2^
- **Shows How:** Competence is demonstrated through structured environments, such as, simulated documentation audits, sandboxed EHRs, or mock policy development for AI-assisted clinical decision support. Rubrics can evaluate equity, safety, compliance, and explainability.^3^
- **Does:** Students engage in real-world, supervised practice—through capstones, practicums, or internships—applying AI in authentic tasks such as coding audits, de-identification reviews, or dashboard evaluations for bias. Assessments may include supervisor evaluations, portfolios, and reflective narratives.^4^

### Modern Extensions of Miller’s Pyramid

Miller’s framework has progressively evolved to address the growing complexity of health professions education, particularly as AI becomes increasingly embedded in health care practice.^8^^,^^9^ This evolution calls for expanded approaches to teaching and assessment that account for emerging ethical, collaborative, and technological demands.

One key extension is the addition of the “Is” dimension—also known as Miller’s Prism—which integrates professional identity, values, and ethical reasoning as core components of competence.^8^ In AI education, this highlights the need for learners to not only develop technical proficiency but also embody responsibility and integrity as ethical stewards of technology in data-driven environments.

Another notable adaptation is the “Do Together” level, which reflects the collaborative reality of AI deployment. AI tools are rarely implemented in isolation; rather, they operate within interdisciplinary teams requiring strong communication and shared decision-making.^9^^,^^10^ Competency, therefore, must be demonstrated in group-based settings, underscoring the need for curricula that foster teamwork and assess performance in collective, technology-rich environments.

Entrustable professional activities (EPAs) offer a third extension by operationalizing the upper levels of Miller’s model. EPAs assess whether learners can be trusted to independently perform complex, high-stakes tasks involving AI—such as reviewing algorithm outputs, conducting documentation audits, or overseeing data governance.^11^ These evaluations go beyond technical ability to evaluate readiness to assume real-world responsibility, including ethical judgment and risk navigation.^9^^,^^12^

Together, these modern extensions enhance Miller’s Pyramid as a framework for building comprehensive, practice-ready AI education. By aligning curricula with these developments, educators can support not only knowledge acquisition but also the development of judgment, identity, and collaborative capacity. This approach equips students to lead responsibly in AI-driven health care and aligns with evolving standards such as the CAHIIM Accreditation Standards and the TIGER Initiative for Informatics Education.^6^^,^^7^

## Designing AI-Enhanced Assignments Using Miller’s Pyramid

**Figure 1. Health Informatics and Information Management AI Framework.**
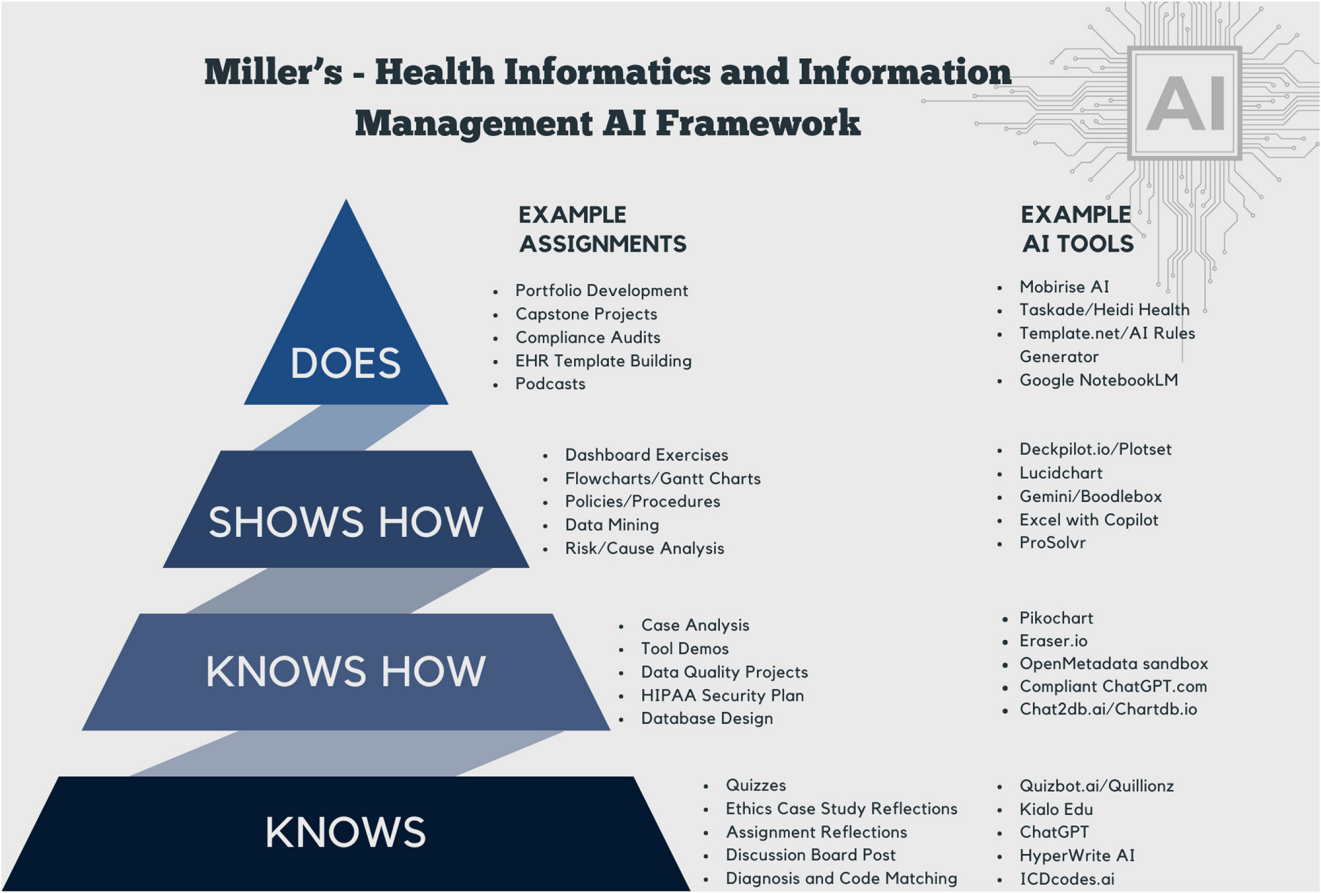
AI, artificial intelligence; EHR, electronic health record; HIPAA, Health Insurance Portability and Accountability Act; ICDCodes, International Classification of Diseases codes.

Integrating AI tools into HIIM education requires more than simply introducing new tools—it calls for structured, pedagogically sound approaches to support skill development. To assist with this, Figure 1 presents the HIIM AI Framework, visually mapping sample assignments and commonly available AI tools to Miller’s Pyramid of Clinical Competence (Knows → Knows How → Shows How → Does). Complementing this, Table 1 outlines aligned learning goals, example assignments, and HIIM-specific contexts for each level of the pyramid. Together, these resources offer a practical and flexible guide for educators designing meaningful, AI-integrated learning experiences.

**Table 1.** AI-Enhanced Assignment Examples Aligned With Miller’s Pyramid and Health Informatics and Information Management Learning Contexts

Miller’s Pyramid Level	Learning Goal	Example Assignments	Example HIIM Contexts
Knows	Recall or define core concepts and terminology related to AI in HIIM.	Quizzes Ethics case study identification Assignment reflections Discussion board post Diagnosis and code matching	Introduction to AI AI definitions and scope in HIIM Coding systems overview Legal and ethical frameworks (HIPAA, etc.)
Knows How	Interpret and apply foundational AI knowledge to HIIM scenarios.	Case analysis Tool demos Data quality projects HIPAA security plan Principles of database design development	Evaluation of AI tool functionality Designing AI-ready databases Privacy assessment using AI models
Shows How	Demonstrate application of AI knowledge in structured or simulated environments.	Dashboard exercises Flowcharts/Gantt charts Mock policies and procedures Data mining Simulated risk/cause analysis	Workflow modeling with AI tools Predictive modeling exercises Mock compliance scenarios
Does	Apply AI-enhanced skills in authentic, real-world HIIM settings.	Portfolio development Capstone projects Compliance audits EHR template building Podcasts	Applied informatics in clinical settings AI-assisted documentation Real-world system evaluations

Abbreviations: AI, artificial intelligence; EHR, electronic health record; HIIM, health informatics and information management; HIPAA, Health Insurance Portability and Accountability Act.

This structured framework enables instructors to tailor assignments to match increasingly complex cognitive and applied skills—ranging from foundational knowledge and conceptual reasoning to simulated application and real-world performance. Each level is situated within an HIIM-specific context to ensure relevance and applicability.

The AI tools included in the framework were carefully selected for their accessibility, intuitive interfaces, and availability in free or low-cost versions—making them feasible for use in a variety of academic settings. These tools not only support technical skill-building but also foster ethical awareness and professional readiness by allowing students to engage directly with contemporary AI applications in health care and information management.

Rather than prescribing a one-size-fits-all solution, the framework offers flexibility—empowering instructors to adapt and innovate assignments based on their own course objectives, institutional priorities, and student learning outcomes. This encourages reflective, intentional integration of AI tools into the curriculum, helping students learn with AI, not just about it, and preparing them to navigate a rapidly evolving digital health landscape with competence and confidence.

## Integrating AI Tools into Hiim Education

Adopting AI tools in HIIM education requires thoughtful planning and the adaptation to an evolving technological landscape. Faculty must navigate a rapidly evolving AI landscape, balancing the opportunities offered by new tools with curricular alignment, usability, and accessibility concerns. As AI technologies are increasingly adopted across health care settings, academic programs must prepare students to understand, evaluate, and apply these tools responsibly and effectively.

To support meaningful adoption, AI tools should align with accreditation and certification standards, enable hands-on learning, and be mapped to competency-based frameworks such as Miller’s Pyramid. This framework supports progressive skill-building—from foundational knowledge to real-world application—across different levels of education.

Table 2 illustrates how AI tools can be integrated into HIIM curricula at the associate, bachelor’s, and graduate levels. Each example aligns learning outcomes with Miller’s Pyramid to support scaffolded, competency-based instruction.

**Table 2.** Adapting AI to Miller’s Framework by Educational Level

Educational Level	Curriculum Area	Example Tools	Description/Use Case (Miller’s Pyramid)
Associate	Introduction to AI in health care	NA	Introduce basic AI (eg, ML, NLP) in health IT or intro to health professions courses (**Knows**)
	AI-assisted coding	ICDcodes.ai, vendor AI coding tools	Practice using AI to automate ICD-10 coding, billing, and claims (**Knows/Knows How/Shows How**)
	Clinical documentation support	Heidi Health	Generate SOAP notes and discharge summaries; assess completeness using AI (**Knows How/Shows How**)
Bachelor’s	Capstone project website	Mobirise AI	Use AI to create websites showcasing capstone projects (**Shows How/Does**)
	Data visualization	PlotSet	Build dashboards from anonymized data to visualize outcomes and trends (**Knows How/Shows How**)
	AI and machine learning	R, RStudio	Analyze datasets and apply ML models (eg, for readmission prediction; **Knows How/Shows How**)
	Governance and compliance	Compliant ChatGPT	Simulate audit reviews, map internal policies to regulatory frameworks (**Knows How/Shows How**)
Graduate	Health analytics	Google Gemini, Google Colab	Perform predictive modeling and strategic decision support with AI tools (**Knows How/Shows How**)
	Machine learning projects	Scikit-learn	Build ML models (supervised/unsupervised) for quality improvement or resource optimization (**Knows How/Shows How/Does**)
	Ethics, governance and policy	Scribbr AI, ChatGPT	Support ethical writing, policy critique, and regulatory analysis in research (**Knows How/Shows How**)

Abbreviations: ML, machine learning; NLP, natural language processing; IT, information technology; ICD-10, International Classification of Diseases, Tenth Revision; SOAP, Subjective, Objective, Assessment, and Plan.

Educators are encouraged to intentionally integrate these tools into assignments, simulations, or capstone activities that reflect real-world health information management scenarios. When used strategically, AI tools can scaffold student learning, bridge the knowing–doing gap, and build longitudinal competence in applied reasoning, technical skills, and ethical decision-making.

For additional support, Appendix A offers instructor-focused guidance for implementing AI-enhanced assignments, offering practical considerations and strategies for effective integration across all academic levels.

## Recommended AI Tools and Resources

Selecting appropriate AI tools for health information education requires thoughtful consideration of usability, accessibility, and alignment with learning outcomes. Cost is a primary factor—educators often seek free or low-cost tools to reduce student burden and promote equitable access. However, free tools may come with limitations in features, data usage, or functionality.

Ease of use is another critical criterion. Tools that are intuitive and require minimal training are ideal for entry-level learners, whereas more advanced platforms may demand technical or domain knowledge, thereby requiring guided tutorials or additional preparation. Accordingly, tools can be categorized as easy or moderate based on their learning curve. Hands-on, repeated use is also essential to support skill development and confidence, especially across different stages of learning.

Beyond technical considerations, tool selection should align with Miller’s Pyramid and its modern extensions. For instance, tools supporting basic exploration and content comprehension are appropriate for the Knows or Knows How stages. In contrast, simulation tools and sandbox environments support Shows How, whereas real-world projects and guided internships align with Does. The “Is” and “Do Together” extensions also invite attention to tools that promote ethical reasoning, collaboration, and professional identity in data-driven environments.

In addition, AI tools can serve as platforms to build digital literacy, introduce students to algorithmic bias, support discussions on privacy and governance, reinforce ethical reasoning, and align with accreditation standards such as those outlined by CAHIIM and the Healthcare Information and Management Systems Society TIGER Initiative. Also, tools that raise awareness about data provenance, reproducibility, and transparency can be especially valuable for fostering critical thinking.

## Ethics of AI-Integrated HIIM Education

As AI becomes increasingly embedded in HIIM education, it brings with it a range of ethical challenges that educators must thoughtfully and proactively address. Teaching technical proficiency alone is no longer sufficient; students must also cultivate competencies in ethical decision-making, equity, and responsible technology use. Key ethical domains relevant to AI integration include data privacy and security, algorithmic bias and fairness, transparency of AI processes, academic integrity in the use of AI-generated content, and equitable access to AI tools. Preparing students to navigate these issues with critical awareness is essential for fostering trustworthy and inclusive health information practices in AI-enabled environments.

To support ethical integration of AI in HIIM education, Table 3 outlines five key ethical domains—Data privacy and security; Algorithmic bias and fairness; Transparency; Academic integrity; and Accessibility and equity—each describing a core problem area and corresponding learning strategies mapped to Miller’s Framework (Knows → Knows How → Shows How→ Does). This scaffolded model guides students from foundational understanding to applied ethical decision‐making in real‐world, AI‐enabled environments.

**Table 3.** Mapping Ethical Issues in AI to Competency-Based Learning Using Miller’s Framework

Ethical Issue	Core Problem Description	Instructional Examples Aligned With Miller’s Framework
Data privacy and security	Uploaded personal or organizational information can be accessed or misused; ownership and storage are unclear	**Knows:** Research AI privacy policies **Knows How:** Debate privacy risks **Shows How:** Draft mock data privacy policies **Does:** Evaluate AI privacy in real projects and confidentiality notices
Algorithmic bias and fairness	Training data may be biased, affecting patient care quality and fairness	**Knows:** Study bias in AI tools **Knows How:** Debate biased outputs **Shows How:** Propose bias mitigation strategies **Does:** Apply fairness principles in practice
Transparency	Lack of clarity on how AI tools are trained or generate outputs	**Knows:** Learn how AI is trained **Knows How:** Debate importance of transparency **Shows How:** Performs explainability analysis using a rubric or simulated tool **Does:** Assess tools for transparency in use
Academic integrity	Students may present AI-generated work as their own, leading to plagiarism	**Knows:** Review citation policies for AI use **Knows How:** Debate misuse scenarios **Shows How:** Evaluate case of improper AI use **Does:** Use AI ethically with documentation
Accessibility and equity	Unequal access or knowledge of AI tools may disadvantage some learners	**Knows:** Explore access and equity issues **Knows How:** Debate use when access is unequal **Shows How:** Analyze mock access challenges **Does:** Audit real-world AI accessibility

### Data Privacy and Security

AI tools often rely on user-submitted data to generate outputs, raising concerns about privacy, ownership, and reidentification risks—even when data are de-identified. Students must understand how to collect, store, and remove information in compliance with institutional policies and professional standards.^13–15^ Integrating case studies and scenario-based discussions can help students apply privacy principles and navigate evolving regulatory landscapes.

### Algorithmic Bias and Fairness

Biases in AI systems—originating from skewed training data or flawed algorithms—can perpetuate inequities in health care. Such bias may reflect demographic or socioeconomic imbalances, leading to unfair or discriminatory outcomes. Students should be trained to identify bias in datasets and outputs and to propose mitigation strategies in both academic and workplace settings.^13^^,^^16^

### Transparency

Transparency in AI system design fosters user trust and accountability. By learning how algorithms function, students can better evaluate whether an AI system’s processes and outputs are explainable, appropriate, and ethically defensible.^17^ Developing this awareness encourages informed decision-making about when and how AI tools should be applied.

### Academic Integrity

The widespread availability of generative AI presents challenges to traditional academic honesty. Misuse—such as submitting AI-generated work as original—undermines learning outcomes and academic credibility. Students should be guided to use AI ethically, cite contributions properly, and maintain independent analytical thinking rather than overrelying on automation.^18^

### Accessibility and Equity

Equitable access to AI tools and learning opportunities is essential. Differences in financial resources, prior technical experience, or disabilities can affect how students engage with AI-based learning environments. Ethical integration requires evaluating cost, usability, and compatibility with assistive technologies to ensure inclusive participation for all learners.^17^

## Discussion

Integrating AI into HIIM education is no longer optional; it is a necessity to ensure graduates are prepared for a data-driven and rapidly evolving workforce. In this article, the authors introduce a pedagogically grounded approach for incorporating AI tools and competencies into curricula by aligning assignment design with Miller’s Pyramid of Clinical Competence. The proposed HIIM AI Framework offers structured guidance for educators by linking AI-enhanced activities with hierarchical learning objectives, promoting both cognitive development and practical application.

Importantly, the framework emphasizes ethics, accessibility, and adaptability. By focusing on not just technical skills but also equity, privacy, academic integrity, and transparency, the model addresses emerging ethical challenges in digital learning. Moreover, by suggesting tools with low-cost or free access, the framework lowers barriers to integration, ensuring more inclusive adoption.

As AI technologies continue to evolve, HIIM educators must remain responsive—revising curricula, collaborating across disciplines, and modeling reflective teaching practices. This framework is not prescriptive but adaptive; its flexibility allows for expansion across different academic levels, institutional types, and learning environments.

At the same time, operationalizing Miller’s Pyramid in HIIM programs presents instructional and structural considerations. Embedding performance-based learning into already dense curricula requires thoughtful curricular mapping, administrative buy-in, and iterative faculty development. Moving from “Knows” to “Does” also calls for access to simulations, applied tools, and novel assessment strategies that go beyond traditional testing.

Integrating AI into this progression adds further opportunities—and responsibilities. Faculty may benefit from additional training and professional development to teach AI confidently, with attention to both technical capability and responsible use. Because AI tools evolve rapidly, ongoing faculty collaboration, knowledge-sharing communities, and institutional support will be essential to keep pace with emerging technologies. Students, too, come with varying levels of digital and data science literacy, so instructional strategies should be scaffolded and inclusive, offering equitable access to learning opportunities.

Despite these implementation needs, aligning AI education with frameworks such as Miller’s Pyramid offers a roadmap for meeting both accreditation requirements (eg, CAHIIM 2026) and workforce readiness. It supports demonstrable competencies across ethical reasoning, technical proficiency, and governance literacy, ensuring learners are not only informed users of AI but also critical thinkers and stewards of digital health systems.

Future directions may include piloting this framework across diverse programs, evaluating learner outcomes, and developing case-based microcredentials or stackable modules that enhance competency in real-world contexts. Institutional investments in faculty development, interdisciplinary collaboration, and digital infrastructure will further enable meaningful integration.

By embracing this structured, flexible, and ethically grounded model, HIIM educators can bridge the knowing–doing gap, cultivate real-world competencies, and prepare future professionals to lead responsibly and confidently in an AI-enhanced health care ecosystem.

## Conclusions

The future of HIIM education lies in the seamless, ethical, and purposeful integration of emerging technologies such as AI. By aligning AI-supported assignments with Miller’s Pyramid and domain-specific competencies, educators can create meaningful, performance-based learning experiences that strengthen technical fluency while advancing professional readiness.

The HIIM AI Framework and accompanying tools presented in this article provide a practical foundation for this integration. Through scaffolded design, ethical grounding, and pedagogical rigor, this approach supports the development of a workforce prepared to navigate complex AI-enabled health care environments with competence, confidence, and critical insight—advancing academic excellence and driving transformation across the HIIM landscape.

## Disclosures

The authors used an AI-assisted writing tool (ChatGPT by OpenAI) between July and December 2025 to support the editing and refinement of manuscript text. The tool was used solely to enhance clarity, structure, and grammar. No AI tools were used for study design, data analysis, or original content generation. The authors take full responsibility for the integrity and accuracy of the manuscript.

## Funding

The authors received no funding for this research.

CE Quiz

## Adapting AI to Miller’s Framework by Educational Level

### Associate Level: Building Foundations

At the associate level, students are introduced to AI concepts and their practical applications in administrative health information tasks such as coding, documentation, and health record management.

Curriculum highlights:

- **Introduction to AI in health care:** Include foundational topics such as machine learning and natural language processing in introductory health IT or applications courses. *(Knows)*
- **Practical AI applications:** Use tools such as *ICDcodes.ai* or vendor-based AI-assisted coding systems for hands-on experience with automated ICD-10 coding, billing, and claims processing. *(Knows/Knows How/Shows How)*
- **Clinical documentation support:** Integrate tools such as *Heidi Health* to practice generating and assessing SOAP notes, discharge summaries, and EHR templates. *(Knows How/Shows How/Does)*

### Bachelor’s Level: Advancing Competency

At the bachelor’s level, students develop a deeper understanding of AI principles, legal and ethical implications, and data analytics. AI can be embedded across the curriculum or offered as a dedicated course.

Curriculum highlights:

- **Capstone project portfolio:** Students can use tools such as *Mobirise AI* to build a portfolio website presenting their capstone project goals, methods, and results. *(Shows How/Does)*
- **Data visualization and analytics:** Tools like *PlotSet* can help students create dashboards to visualize patient outcomes, quality measures, or health trends using real or anonymized datasets. *(Knows How/Shows How)*
- **AI and machine learning:** Use *R* and *RStudio* to analyze publicly available datasets and apply basic machine learning models (eg, hospital readmission prediction). *(Knows How/Shows How)*
- **Governance and compliance:** Explore AI-related legal, privacy, and ethical issues in health information through tools like *Compliant ChatGPT* to simulate audits or compliance mapping. *(Knows How/Shows How)*

### Graduate Level: Leading With Strategy and Ethics

Graduate students should focus on advanced technical applications, leadership in AI adoption, and ethical and policy considerations in health systems.

Curriculum highlights:

- **Health analytics with AI:** Tools such as *Google Gemini* and *Google Colab* can be used for predictive modeling, data visualization, and strategic decision-making. *(Knows How/Shows How)*
- **Machine learning proficiency:** Advanced projects can use *Scikit-learn* for supervised/unsupervised learning models to inform quality improvement or resource optimization. *(Knows How/Shows How/Does)*
- **Ethics, governance, and policy:** Tools such as *Scribbr AI* and *ChatGPT* support research writing and critical evaluation of AI ethics, policy development, and regulatory frameworks. *(Knows How/Shows How)*
